# Text-Book of General Pathology

**Published:** 1913-12

**Authors:** 


**Text-Book of General Pathology.** Edited by M. S. PEMBREY and J. RITCHIE. Pp. xii, 773. London: Edward Arnold. 1913. Price 18s. net.

The term General Pathology through usage has become associated in the minds of the profession with something very different in detail if not in principle from the exposition of the subject provided in this book, and it seems to us unfortunate that in endeavouring to awaken the profession to a true conception of Pathology, the editors have agreed to accept the old-established title associated so much in the past with a study of morbid processes. The title "Applied Physiology," or more correctly "Clinical Applied Physiology," would have been very suitable, and would have indicated the scope of the text and assisted in introducing the volume to those for whom in our opinion it is a necessity, the practising physician and surgeon, and students reading for research and the higher qualifications.

The authors of the many articles have covered the ground thoroughly, and there is astonishingly little repetition or overlapping. As is to be expected, the different writers vary much in their power to sustain the interest of the reader in their subjects; nevertheless there are few dull pages in the book, and bearing in mind that they have set out as pioneers of a wider knowledge and understanding of Physiology and Pathology, they are to be congratulated on the lucidity of their exposition of subjects that are often obscured by technicalities, and on avoiding the pitfall of hypothesis and theory, as useless to the clinician as they are valuable to the investigator.

The book ought to be in the hands of every medical man who cares to advance with the times, and on the laboratory shelf of those who desire to lead the van of progress.

